# Timings of pre-hospital life-saving interventions in mass casualty incidents: an observational simulation study

**DOI:** 10.1186/s13049-025-01417-z

**Published:** 2025-06-02

**Authors:** Fayez Alruqi, Elaine Cole, Karim Brohi

**Affiliations:** 1 Centre for Trauma Sciences, Blizard Institute, Queen Mary University of London, 4 Newark St, London, E1 2AT UK; 2 Emergency Medical Services Program, Department of Nursing, College of Nursing and Health Sciences, Jazan University, Al Maarefah Rd, Jazan, 45142 Saudi Arabia

**Keywords:** Pre-hospital care, Life-saving intervention, Time, Simulation, Trauma, Mass casualty incident

## Abstract

**Background:**

Mass casualty incidents (MCIs) pose significant challenges for pre-hospital care. In particular, there is a tension between the need for rapid triage and the need to deliver life-saving interventions (LSIs). Currently, only the simplest interventions are considered appropriate during triage. However, few data exist on how long it takes to perform LSIs, and there may be a difference between perception and reality. This study aims to determine the time intervals (TIs) to perform key LSIs in a simulated pre-hospital setting, and the differences between estimated and actual TIs.

**Methods:**

An observational simulation study was conducted over three sessions at two pre-hospital training centers. Pre-hospital care providers (PHCPs) performed 16 LSIs. A pre-intervention questionnaire was used to assess the participants’ backgrounds and LSI experience. Non-parametric tests were used to compare TIs between professional groups and evaluate differences between estimated and actual TIs.

**Results:**

Twenty PHCPs participated: eight physicians and 12 paramedics, with a median pre-hospital experience of nine years. TIs for all LSIs were ≤ 130 s, except for rapid sequence induction and intubation (RSI), median 348 s (IQR: 329–366). Team-based LSIs where paramedics and physicians worked together, had prolonged durations for certain steps, with the RSI preparation stage being the longest (59% of total TI). Considerable delays were also observed in the post-placement securing phase (RSI: 43 s, chest tube: 58 s). All PHCPs tended to overestimate how long TIs take, with significant differences noted in supraglottic airway insertion, cricothyroidotomy, needle decompression and finger thoracostomy.

**Conclusion:**

We provide data on the time taken for LSIs in a simulated pre-hospital environment. Nearly all LSIs were completed within two minutes, yet PHCPs overestimated the time it takes to perform them. Approaches to the triage process may need to be reviewed in light of these data. There are further opportunities to streamline the delivery of some interventions.

**Supplementary Information:**

The online version contains supplementary material available at 10.1186/s13049-025-01417-z.

## Background

Mass casualty incidents (MCIs) have caused over 1.6 million deaths and countless injuries worldwide in the past two decades [1]. Most fatalities occur on-scene in the pre-hospital setting, where early life-saving interventions (LSIs) could prevent many deaths [2]. However, MCI triage systems prioritize speed, allowing basic LSIs like manual airway opening or direct pressure for external hemorrhage [3, 4], while other essential LSIs, including tourniquet application or needle decompression, are omitted [5]. This exclusion is primarily based on the assumption that LSIs prolong on-scene time and divert resources away from high-priority casualties [6]. Yet, without empirical data on LSI timings, triage decisions rely on unsupported assumptions. This gap risks unnecessary delays and the omission of critical interventions, jeopardizing patient survival.

During MCIs, prioritizing triage over delivering essential LSIs can have severe consequences. Recent incidents such as the 2017 Manchester Arena bombing exposed significant delays in providing early LSIs as pre-hospital care providers (PHCPs) focused on triaging other victims [7]. These delays resulted in preventable deaths, highlighting a critical gap in pre-hospital care [7]. Previous MCIs have prompted policymakers and researchers to advocate for improvements to triage systems, including the integration of a broader range of interventions [7, 8]. However, current triage tools, including newly developed systems designed to address care gaps, inconsistently include LSIs, leaving many critical interventions unused due to perceived time constraints [5, 9]. For example, interventions such as oral fentanyl and intramuscular (IM) tranexamic acid (TXA), both of which have been shown to be rapid and effective, are often ignored in both standard and MCI scenarios despite their potential to improve outcomes [8, 10]. These inconsistencies not only hinder standardization across global MCI responses but also raise concerns about which LSIs can be safely integrated into triage without delaying care.

The overall objective of this study was to identify the average time intervals (TIs) required for performing different LSIs in a pre-hospital simulated setting, including stepwise time points for team-based interventions. We specifically aimed to measure the TIs for basic and advanced LSIs, compare the performance between paramedics and physicians, and examine the discrepancies between estimated and actual TIs to identify patterns of overestimation or underestimation.

## Methods

### Study design and setting

A prospective simulation study was conducted at London’s Air Ambulance (LAA) and Essex & Herts Air Ambulance (EHAAT) training centers. From July to November 2023, pre-hospital care providers (PHCPs), were invited to participate in three simulation sessions to measure the timings to perform pre-hospital LSIs which may be used in an MCI. Reporting adhered to the STROBE (Strengthening the Reporting of Observational Studies in Epidemiology) statement, utilizing the specific extensions for healthcare simulation research (Supplementary Material, Table A) [11].

### Participant recruitment and informed consent

PHCPs including paramedics and physicians across a range of pre-hospital expertise levels, without specific requirements for years of service, were invited to participate. PHCPs received an email about the study via their respective pre-hospital services/institutes and were provided with information about the objectives and procedures of the simulation sessions. The participant information sheet, outlining the study’s aims and ethical considerations, was distributed ahead of the sessions to ensure informed consent. Prior to participating in the simulation, participants were asked to sign a consent form.

### Study sample

No formal sample size was calculated and the study used a convenience sample of PHCPs who were willing to participate in these sessions and were made available by their pre-hospital services.

### Procedures

Participants performed standard care procedures for adult mixed-fidelity simulated patients in non-mass casualty incident scenarios. Participants entered the simulation sessions in pairs (paramedic and physician), but each performed most of the LSIs individually. Advanced LSIs such as rapid sequence induction and intubation (RSI), chest tube insertion, and cricothyroidotomy required assistance from another participant to mirror “real-world” team-work practice. Other LSIs including finger thoracostomy, intravenous (IV) access, and application of direct pressure were captured as components of broader procedures like chest tube insertion, fluid resuscitation, and hemostatic agent application, respectively. PHCPs were restricted to performing only those LSIs that fell within the scope of their practice as defined by their respective professional roles. A standardized RSI checklist was used during the simulation to facilitate consistent equipment preparation, team briefing, and procedural readiness across participants (Supplementary Material, Figure A).

### Equipment

The mannequins utilized in the simulations were SIMBODIES Manikin^®^ (Advanced Adult Manikin) and Laerdal Manikin^®^ (Crash Kelly) & (Extri Kelly) serving as life-like patient simulators for all interventions. Specific equipment required for each intervention and guidelines are detailed in (Supplementary Material, Table B).

### Outcome measure

The primary outcome of the study was the Time Interval (TI)—the period from when the participant touched the equipment or mannequin to the completion of the intervention— for 16 LSIs that were pre-identified through a literature review as frequently performed interventions in mass casualty pre-hospital care (Table 1).

**Table 1:** Interventions and corresponding end points for life-saving interventions*

Intervention		End Point
Airway interventions	Oropharyngeal airway	Complete first Bag-valve-mask (BVM) ventilation
	Supraglottic airway (i-gel)	
	Cricothyroidotomy	
	Rapid sequence induction and intubation	Tube is secured and BVM ventilation is delivered
Breathing interventions	Oxygen (non-rebreather mask)	Secure face mask with proper oxygen flow
	Needle decompression	Remove needle to sharp container
	Finger thoracostomy	Finger withdrawn from incision
	Chest tube	Tube is securely sutured and dressed
Circulation interventions	Direct pressure	The gauze is placed over the wound and pressure applied
	Hemostatic agent (Celox^®^)	Bandage clipped after wound wrapping
	Tourniquet (CAT)	Completely secure on the limb
	Intramuscular Tranexamic acid	Remove needle to sharp container
	Intraosseous access	Secure the line and remove needle to sharp container
	Intravenous access	
	Fluid resuscitation	Open drip chamber after IV tube insertion
	Oral fentanyl	Placed in the oral cavity with the drug matrix moved from one side to the other, and the simulated patient instructed to suck

*Commencement of interventions is defined as the moment the equipment is picked up or the mannequin is touched

Further time point descriptors were as follows:

- A) Rapid sequence induction and intubation (RSI)
  - *Administer induction agents*: marked by the start of the syringe’s bolus push.
  - *Visualize vocal cords*: when the participant either verbally confirms the visibility of the vocal cords or requests a bougie.
  - *Insert endotracheal tube*: the tube passing the teeth level as it enters the mouth.
  - *Deliver first ventilation*: successful delivery of the first ventilation via BVM.
- B) Cricothyrotomy
  - *Skin incision*: moment the scalpel touches and moves on the skin to make an incision.
  - *Insert endotracheal tube*: when the cricothyrotomy tube passes the skin level into the trachea.
  - *Delivered first ventilation*: successful delivery of the first ventilation via BVM.
- C) Chest tube 
  - *Skin incision*: moment the scalpel touches and moves on the skin to make an incision.
  - *Place finger*: moment the finger begins to penetrate the incision site
  - *Complete finger thoracostomy*: marked by the complete removal of the finger from the incision.
  - *Place the chest tube*: moment the tip of the chest tube is inserted into the incision.
  - *Start suturing*: identified when the metal suture needle penetrates the skin.
  - *Intervention completed*: tube is securely sutured and dressed.

### Data collection process

After providing consent to participate, a pre-intervention questionnaire (Supplementary Material, Figure B) was distributed to collect participants’ professional backgrounds and their perceptions of the LSIs’ importance in MCIs. Additionally, participants were asked to estimate the time required for each LSI in Table 1, to gather their perception of expected intervention durations. These estimated times were subsequently defined as the “expected timings” for comparison with the timings observed during the simulation. The participants were asked to confirm which of the 16 pre-defined LSIs they were certified to perform within their scope of practice and those they had performed in their service roles.

Participants’ performances of completing LSIs were video and audio recorded using a GoPro Hero 10 camera (GoPro, San Mateo, CA, USA). The recorded interventions were stored on SD memory cards, then transferred to a secure computer with password protected access to ensure the confidentiality of the data at each stage of handling. Adobe Premiere Pro Version 24.1 software (Adobe, San Jose, CA, USA) was used for the analysis, and this allowed the TI and unique time points for team-based interventions to be accurately captured. The raw data from capture times and questionnaire were exported to Excel software Microsoft Excel 2023 (Version 16.79.1, Redmond, WA, USA) prior to data analysis.

### Data analysis

Statistical data analyzing was performed using RStudio 2023.9.1.494 (RStudio, Boston, MA, USA). Participant demographics were analyzed using frequencies and percentages. Normality testing for continuous data was performed using the Shapiro–Wilk test. The results indicated that some data appeared to follow a normal distribution. However, due to the low sample size, the reliability of these tests is limited [12]. Visual inspection of histograms further suggested deviations from normality for most variables. Therefore, the Mann–Whitney U test was utilized to compare TIs for each LSI across two groups: paramedics and physicians and findings presented as median and interquartile range (IQR). Wilcoxon Signed-Rank test was used to evaluate the differences between the participant estimated and actual TI for each intervention. All recorded TIs were presented in seconds and rounded to the nearest whole number for clarity. All tests were two tailed with the results considered statistically significance if the *p* value was < 0.05.

### Ethical considerations

The ethics committee of Queen Mary University of London approved the study protocol (Approval ID: QMERC23.097).

## Results

Twenty PHCPs participated in three simulation sessions, which included eight physicians (40%) and 12 (60%) paramedics (Table 2). The participants median pre-hospital work experience was 9 years (IQR: 3–14), and seven (35%) had prior experience of responding to MCIs. A total of 246 LSIs were performed, with interventions restricted by the scope of practice (Supplementary Material, Figure C). RSI and chest tube insertion were performed only by physicians or advanced paramedics. Additionally, oral transmucosal fentanyl citrate (OTFC) administration was limited to advanced PHCPs in one air ambulance service.

**Table 2:** Participants demographics and performed life-saving interventions characteristics in simulation sessions

	**Paramedic** **(Median [IQR] or N)**	**Physician** **(Median [IQR] or N)**	**Total** **(Median [IQR] or N)**
Number of participants	12	8	20
Previous experience			
- Years of experience	14 (IQR: 9–19)	2 (IQR: 1–6)	9 (IQR: 3–14)
- Participants with Prior MCI Experience	5	2	7
Number of LSIs performed during simulation sessions			
- Airway management LSIs	33	29	62
- Breathing support LSIs	28	27	55
- Circulation support LSIs	78	51	129
- Total LSIs	139	107	246

*Abbreviations*: *IQR* Interquartile range, *LSI* Life-saving intervention, *MCI* Mass casualty incident, *N* Number

### Intervention timings across participants

**Fig. 1:**
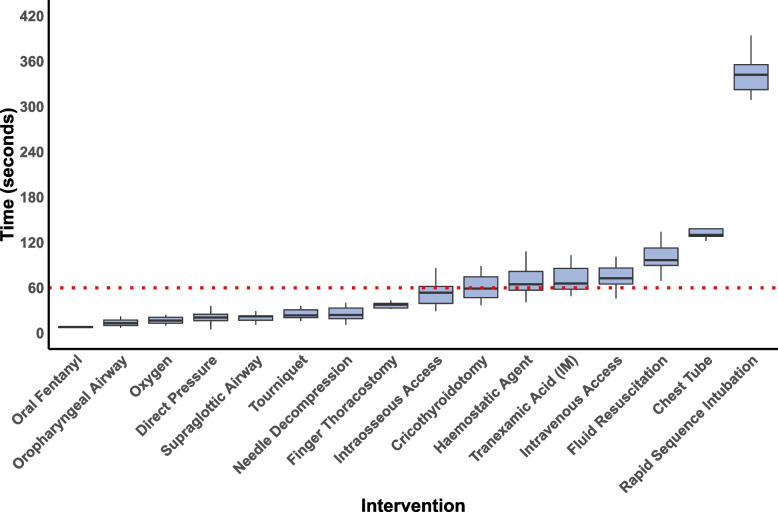
Box and whisker plot illustrating time taken in seconds for different life-saving interventions* The boxes represent the interquartile range (IQR), which contains the middle 50% of the data, while the horizontal lines inside the boxes represent the median values. The “whiskers” extend to the smallest and largest values within 1.5 times the IQR from the first and third quartiles, respectively. Outliers have been excluded to enhance interpretability *A dotted horizontal line is drawn at the 1-min mark, representing the threshold where most interventions are deemed suitable for delivery during triage

All LSIs, with the exception of RSI, were performed within a median TI below 2.2 min (132 s), the highest of which was observed for chest tube insertion at 130 s (IQR: 128–138) (Fig. 1). RSI had the longest TI among all interventions, with a median of 348 s (IQR: 329–366), whilst oropharyngeal airway (OPA) placement and OTFC administration were the fastest, with median times of 13 s (IQR: 10–17) and 8 s (IQR: 8–9), respectively. LSIs with a median time exceeding 30 s were generally characterized by substantial variability in timings, except for finger thoracostomy (Fig. 1).

**Fig. 2:**
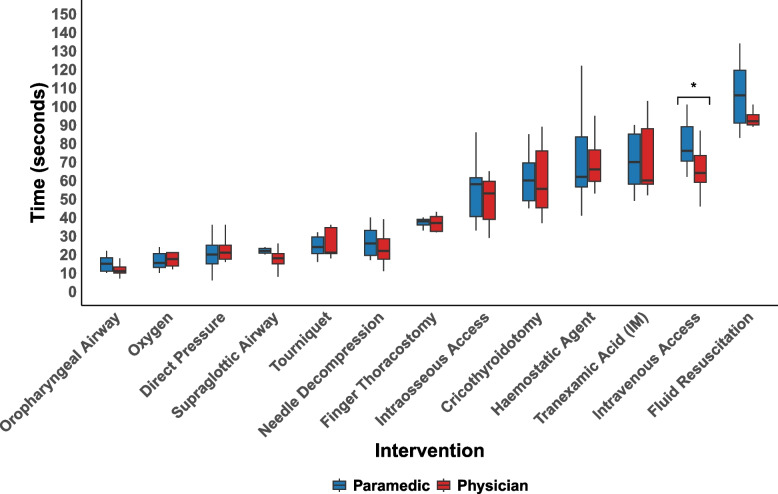
Box and whisker plot of intervention times by professional group The details of the box plots are as described in Fig. 1 * Indicates a statistically significant difference (*p* < 0.05) in the performance times for IV access between paramedics and physicians, as determined by the Mann–Whitney U test

Physicians were faster than paramedics across many interventions except applying hemostatic agents, applying direct pressure, and administering oxygen (Fig. 2). There were no significant differences in LSI TIs between paramedics and physicians, except for IV access, (Physicians 64 s (IQR: 59–74) vs. Paramedics 76 s (IQR: 71–89), *p* = 0.04) (Fig. 2).

There was a general trend to overestimation in TIs for each LSI, with actual times often exceeding estimates (Table 3). This discrepancy varied across different procedures, with some showing a closer alignment between estimated and actual times, while others exhibited significant variances. Notably, supraglottic airway insertion (i-gel) was estimated at a median of 40 s (IQR: 20–60) while the actual median was 22 s (IQR: 17–23) (*p* = 0.002), finger thoracostomy showed a median estimated time of 60 s (IQR: 53–98) against an actual median of 38 s (IQR: 33–39) (*p* = 0.02). Cricothyroidotomy had a median estimated time of 120 s (IQR: 60–190) versus an actual median of 59 s (IQR: 47–75) (*p* = 0.02) and needle decompression had a median estimated time of 30 s (IQR: 20–60) compared to an actual median time of 24 s (IQR: 19–33) (*p* = 0.04).

**Table 3:** Observed and estimated time intervals for life-saving interventions performed in the simulation sessions

Intervention Category	Intervention	Observed TI (seconds) Median (IQR)	Estimated TI (seconds) Median (IQR)	Estimation Direction	*P*-value^*^
Airway interventions	Oropharyngeal airway	13 (IQR: 10–17)	10 (IQR: 10–28)	Underestimated	0.54
	Supraglottic airway (i-gel)	22 (IQR: 17–23)	40 (IQR: 20–60)	Overestimated	**0.002**
	Cricothyroidotomy	59 (IQR: 47–75)	120 (IQR: 60–190)	Overestimated	**0.02**
	Rapid sequence intubation	348 (IQR: 329–366)	390 (IQR: 285–600)	Overestimated	1
Breathing interventions	Oxygen (non-rebreather mask)	17 (IQR: 13–21)	15 (IQR: 10–28)	Underestimated	0.69
	Needle decompression	24 (IQR: 19–33)	30 (IQR: 20–60)	Overestimated	**0.04**
	Finger thoracostomy	38 (IQR: 33–39)	60 (IQR: 53–98)	Overestimated	**0.02**
	Chest tube	130 (IQR: 128–138)	300 (IQR: 150–600)	Overestimated	0.63
Circulation interventions	Direct pressure	21 (IQR: 17–25)	15 (IQR: 9–30)	Underestimated	0.63
	Hemostatic agent (Celox^®^)	65 (IQR: 57–82)	60 (IQR: 40–75)	Underestimated	0.31
	Tourniquet (CAT)	24 (IQR: 20–31)	30 (IQR: 20–53)	Overestimated	0.23
	Intramuscular Tranexamic acid	66 (IQR: 58–86)	60 (IQR: 30–90)	Underestimated	0.38
	Intraosseous access	54 (IQR: 39–62)	60 (IQR: 43–150)	Overestimated	0.74
	Intravenous access	73 (IQR: 65–86)	60 (IQR: 33–105)	Underestimated	0.14
	Fluid resuscitation	97 (IQR: 90–113)	120 (IQR: 60–300)	Overestimated	0.25
	Oral fentanyl	8 (IQR: 8–9)	27 (IQR: 20–53)	Overestimated	0.25

*Abbreviations*: *IM* intramuscular access, *IQR* Interquartile range, *TI* Time Interval

^*^Wilcoxon test used to compare observed and estimated intervention times. It is important to note that some interventions may have incomplete paired data due to restrictions on certain participants performing these interventions, based on professional qualifications or service-specific protocols. This has implications for the presence and interpretation of significance markers, particularly in cases where estimated times are provided without corresponding actual performance data

### Team-based intervention timings

**Fig. 3:**
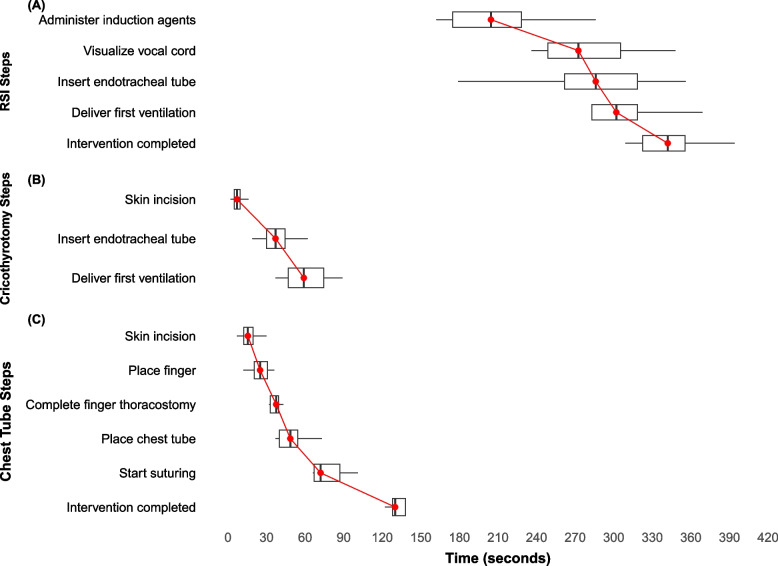
Box and whisker plots illustrate the times for each step in team-based intervention Covers subfigures: **A** Rapid Sequence Intubation (RSI), **B** Cricothyrotomy, and **C** Chest tube placement*. The details of the box plots are as described in Fig. 1 *The last three steps in the chest tube procedure (Place chest tube, start suturing, and intervention completed) were performed only by physicians, as they are the only providers certified to carry out these specific interventions

The median time in team-based LSIs’ steps increased steadily across the initial steps with relatively uniform intervals (Fig. 3). However, the RSI preparation phase, occurring prior to the administration of induction agents, was the longest interval across all team-based interventions assessed, with a median of 205 s, representing 58.8% of the total time for RSI (Fig. 3A). A notable time gap was observed in the post-placement securing phase, with a median time of 43 s for securing the endotracheal tube in RSI and a median of 58 s for suturing in the chest tube procedure (Figs. 3A, C).

## Discussion

This study comprehensively examines different LSI timings, expanding beyond previous literature primarily focused on single LSIs [8, 10]. Nearly all LSIs were completed within two minutes, except for RSI, which had a median duration of just under six minutes. Differences in TI across PHCP qualifications were minimal, however, PHCPs generally overestimated TIs with significant discrepancies observed in i-gel, finger thoracostomy, cricothyroidotomy, and needle decompression. In team-based LSIs, captured step durations increased steadily, with RSI preparation being the longest phase. Additionally, notable delays were observed in securing procedures, particularly for RSI and chest tube insertion.

Our findings show that interventions like oral fentanyl, OPA, oxygen delivery, and i-gel can be performed in similar or significantly less time compared to triage LSIs [5]. Integrating these faster interventions into triage protocols could reduce on-scene mortality and ensure timely critical care. OTFC emerged the fastest intervention in our study and has recently been adopted into civilian pre-hospital settings from military practices [10]. Given its rapid application, OTFC utilized to meet the need for rapid intervention and prevent care gaps in MCIs [10]; however, its adoption remains limited to specific regions, with London's Air Ambulance among the few implement it. Similarly, OPA and i-gel, which are both quick to perform and have high success rates, could be feasible for bystander use in MCIs [13]. The i-gel has the fastest insertion timings among airway devices, even when used with chemical, biological, radiological, and nuclear (CBRN) protective equipment, which supports its use in high-stress, resource-limited settings [14]. Similarly, other rapid LSIs, such as OTFC or OPA, could be integrated into initial response algorithms or tiered approaches to enable timely care by a wider range of responders and reduce scene delays.

RSI had the longest TI, taking 218 s longer than the chest tube, the second-longest procedure. Southard et al. reported a longer RSI time of 479 s—131 s more than our study’s median of 348 s—likely due to differences in measurement criteria [15]. Southard et al. define TI from the moment the flight crew crossed the ambulance threshold until the patient was declared transport-ready, whereas we recorded from equipment pickup or initial mannequin contact until airway security was achieved. Vincent-Lambert et al. reported a median RSI time of 14 min and 2 s; however, this included post-induction sedation and was performed by pre-hospital care students [16]. Nevertheless, RSI is rarely performed in MCIs due to its complexity, which strains limited resources by requiring advanced and multiple PHCPs as well as specialized equipment [17, 18]. Additionally, RSI requires continued airway management, which is challenging when triage is ongoing, and other patients need immediate care. Therefore, this substantial time and resource investment underlines the need for MCI pre-hospital care protocols to carefully evaluate the threshold for performing RSI. Strategies to reduce unnecessary delays may include standardized equipment kits with pre-packed components and pre-assigned team roles.

PHCPs must fully assess the time required to perform LSIs, carefully weighing the risks, benefits, and the urgent need for such interventions. Maintaining situational awareness enhances time perception, reducing delays and improving outcomes [19]. However, estimating LSI TIs remains challenging due to the unpredictable pre-hospital environment. Overestimation of LSI TIs may be attributed to the stress and high-pressure MCI environment in which they operate, known as distortion of time [19]. Studies have shown that during high-arousal or life-threatening situations, such as MCIs, individuals often perceive time as slowing down [19, 20]. Consequently, PHCPs in this study may have believed interventions take longer than they actually did due to this altered perception of time. This discrepancy highlights the value of immersive training environments that mimic MCI-related stress, which could help PHCPs to calibrate their time perception and improve decision-making under pressure [21].

Advanced invasive LSIs consume valuable resources and can delay on-scene time, potentially worsening patient outcome [22]. This study found RSI had prolonged preparation phases 58.8% of TI, lasting longer than the actual tube insertion itself. While preparation is necessary, optimizing certain aspects could reduce delays. Vincent-Lambert et al. similarly reported significant preparation time, though direct comparisons are limited by differing phase definitions [16]. We defined the preparation phase as the period before the syringe’s bolus push, aligning with findings from previous studies where preparation and pre-oxygenation constituted a major proportion of TI, highlighting the time-intensive nature of these early stages [16]. The securing phase also showed delays, particularly in RSI and chest tube procedures. Chest tube suturing had a median time of 58 s, shorter than the 96.3 ± 29.0 s reported by Mckee et al. but still identifying areas of delay [23]. RSI tube-securing delays may stem from the standard use of tube ties, suggesting that transitioning to tube holders could reduce TI [24]. To incorporate advanced LSIs into future MCI protocols, efforts should focus on minimizing unnecessary preparation and post-placement delays.

### Limitation

This study has several limitations. The main limitation is that the study was conducted in a simulated environment, devoid of real-world distractions, consequences, or risks. Despite the use of advanced simulation equipment, the setting may not fully replicate the complexity, unpredictability, and immediacy of large-scale real-life emergency incidents. Furthermore, real-world emergencies present unpredictable variables, including patient characteristics geographical location or incident type, which can influence the timing and feasibility of interventions. Secondly, despite efforts to standardize interventions, the complexity of advanced LSIs across different crews challenges the replication of identical timing trajectories, contributing to observed discrepancies in captured time points. Finally, the convenience sample, limited to available PHCPs, may restrict generalizability. A larger sample would likely offer a more representative overview of clinician experiences.

## Conclusion

Most LSIs were completed in less than one minute, with no significant TI differences between paramedics and physicians. While RSI required more time, many other interventions were performed as fast as those already included in MCI triage tools. However, PHCPs overestimated most LSI TIs, which may have reinforced concerns about triage delays, contributing to the selective incorporation of LSIs in MCI protocols. Future research could incorporate these findings into computer models to optimize MCI response strategies, addressing the limitations of anecdotal studies and simulation exercises in replicating real-world scenarios.

## Supplementary Information

Supplementary Material 1.

## Data Availability

No datasets were generated or analysed during the current study.

## Abbreviations

BVM: Bag-Valve-Mask
EHAAT: Essex & Herts Air Ambulance
IM: Intramuscular
IQR: Interquartile Range
IV: Intravenous
LAA: London’s Air Ambulance
LSI: Life-Saving Interventions
MCI: Mass Casualty Incident
OPA: Oropharyngeal Airway
OTFC: Oral Transmucosal Fentanyl Citrate
PHCP: Pre-Hospital Care Provider
RSI: Rapid Sequence Induction and Intubation
STROBE: The Strengthening the Reporting of Observational Studies In Epidemiology
TI: Time Intervals
TXA: Tranexamic Acid
