# Supplementary material for: Timings of pre-hospital life-saving interventions in mass casualty incidents: an observational simulation study
Source: Scand J Trauma Resusc Emerg Med. 2025 Jun 2;33:100. doi: 10.1186/s13049-025-01417-z (PMC12131418; doi:10.1186/s13049-025-01417-z)
Supplement: Supplementary file 1 — Supplementary Material 1. [file 13049_2025_1417_MOESM1_ESM.zip › Supplementary Material, Figure A.docx]

Figure A: Pre-RSI challenge-response checklist
