# Supplementary material for: Timings of pre-hospital life-saving interventions in mass casualty incidents: an observational simulation study
Source: Scand J Trauma Resusc Emerg Med. 2025 Jun 2;33:100. doi: 10.1186/s13049-025-01417-z (PMC12131418; doi:10.1186/s13049-025-01417-z)
Supplement: Supplementary file 1 — Supplementary Material 1. [file 13049_2025_1417_MOESM1_ESM.zip › Supplementary Material, Figure C.docx]

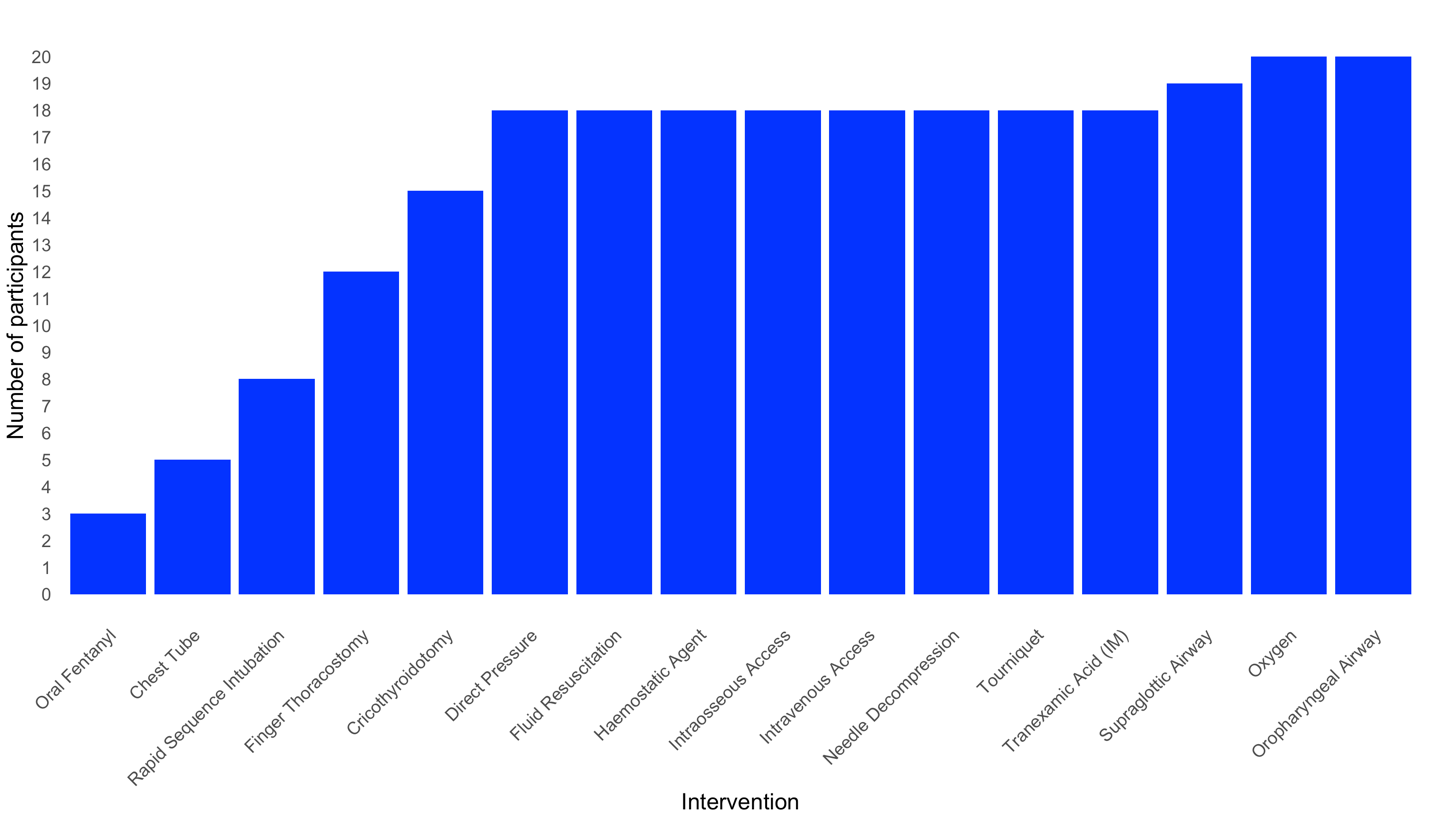


Figure C: Bar graph illustrating the number of participants who performed each type of intervention
