# Supplementary material for: Timings of pre-hospital life-saving interventions in mass casualty incidents: an observational simulation study
Source: Scand J Trauma Resusc Emerg Med. 2025 Jun 2;33:100. doi: 10.1186/s13049-025-01417-z (PMC12131418; doi:10.1186/s13049-025-01417-z)
Supplement: Supplementary file 1 — Supplementary Material 1. [file 13049_2025_1417_MOESM1_ESM.zip › Supplementary Material, Table A.docx]

Table A: STROBE Statement Extension for Simulation-Based Research (STROBE-Sim) Checklist

| **Checklist Item** | **Section** | **STROBE Description (Observational Studies)** | **Extension for SBR** | **Location in Manuscript / How Addressed** |
| --- | --- | --- | --- | --- |
| 1 | Title and abstract | (a) Indicate the study’s design with a commonly used term in the title or the abstract.  (b) Provide in the abstract an informative and balanced summary of what was done and what was found. | In abstract or key terms, the MESH or searchable keyword term must have the word simulation or simulated. | **Addressed:**  -Title includes “observational simulation study”, Page 1.  - Abstract includes “Perform key LSIs in a simulated pre-hospital setting”, Page 2.  - Keywords include “simulation”, Page 3. |
|  | **Introduction** |  |  |  |
| 2 | Background/rationale | Explain the scientific background and rationale for the investigation being reported. | Clarify whether simulation is subject of research or investigational method for research. | **Addressed:** Background section details MCI challenges, triage limitations, and the need for LSI timing data. Simulation is clearly stated as the investigational method used. (Background section), Page 5. |
| 3 | Objectives | State specific objectives, including any prespecified hypotheses. | - | **Addressed:** Objectives stated: “identify the average time intervals (TIs) required for performing different LSIs… measure the TIs for basic and advanced LSIs, compare the performance between paramedics and physicians, and examine the discrepancies between estimated and actual TIs…”. (End of Background section), Page 5. |
|  | **Methods** |  |  |  |
| 4 | Study design | Present key elements of study design early in the paper. | - | **Addressed:** Described as a “prospective simulation study” and “observational simulation study”. (Methods: Study Design and Setting section), Page 5. |
| 5 | Setting | Describe the setting, locations, and relevant dates, including periods of recruitment, exposure, follow-up, and data collection. | - | **Addressed:** Conducted at “London’s Air Ambulance (LAA) and Essex and Herts Air Ambulance (EHAAT) training centers” from “July to November 2023”. Data collected during simulation sessions. (Methods: Study Design and Setting section), Page 5 |
| 6 | Participants | (a) Cohort study: Give the eligibility criteria, and the sources and methods of selection of participants. Describe methods of follow-up. Case-control study: Give the eligibility criteria and the sources and methods of case ascertainment and control selection. Give the rationale for the choice of cases and controls. Cross-sectional study: Give the eligibility criteria and the sources and methods of selection of participants. (b) Cohort study: For matched studies, give matching criteria and number of exposed and unexposed. Case-control study: For matched studies, give matching criteria and the number of controls per case. | - | **Addressed:**  - Participants: Pre-hospital care providers (PHCPs) - paramedics and physicians. (Methods: Participant recruitment and informed consent, Page 5)  - Eligibility: PHCPs across a range of pre-hospital expertise levels. (Methods: Participant recruitment and informed consent, Page 5)  - Selection: Convenience sample via email invitation through services/institutes. (Methods: Participant recruitment and informed consent, Page 5)  - 20 participants included (12 paramedics, 8 physicians). (Results sections, Table 2) |
| 7 | Variables | Clearly define all outcomes, exposures, predictors, potential confounders, and effect modifiers. Give diagnostic criteria, if applicable. | Describe the theoretical and/or conceptual rationale for the design of the intervention/exposure. Describe the intervention/exposure with sufficient detail to permit replication. Clearly describe all simulation-specific exposures, potential confounders, and effect modifiers. | **Addressed:**  - Primary Outcome: Time Interval (TI) for 16 LSIs (defined in Table 1).  - Intervention, Exposure: Performance of specific LSIs. (Methods: Procedures, Page 6).  - Rationale: LSIs identified via literature review. (Methods: Outcome Measure, Page 6-7)  - Simulation-specific exposures:  Standardized procedures, mannequins, equipment (detailed in Supp. Table B), RSI checklist used. (Methods: Procedures, Page 6).  - Potential confounders/modifiers: PHCP qualification, years of experience, prior MCI experience. |
| 8 | Data sources/measurement | For each variable of interest, give sources of data and details of methods of assessment (measurement). Describe comparability of assessment methods if there is >1 group. | In describing the details of methods of assessment, include (when applicable) the setting, instrument, simulator type, timing in relation to the intervention, along with any methods used to enhance the quality of measurements. Provide evidence to support the validity and reliability of assessment tools in this context (if available). | **Addressed:**  - Data Source: Video/audio recordings (GoPro Hero 10).  (Methods: Data Collection Process, Page 8).  - Measurement: TI timed using Adobe Premiere Pro from equipment/mannequin touch to completion (defined per LSI in Table 1). (Methods: Data Collection Process, Page 9).  - Setting: Simulation centers.  Instrument: GoPro, Adobe Premiere Pro. (Methods: Data Collection Process, Page 8-9)  Simulator Type: SIMBODIES Manikin®, Laerdal Manikin® (Crash Kelly & Extri Kelly). (Methods: Equipment, Page 6)  - Quality Enhancement: Standardized procedures, RSI checklist. (Methods: Procedures, Page 6).  - Validity/Reliability: Not applicable, but standard equipment/procedures used. |
| 9 | Bias | Describe any efforts to address potential sources of bias. | - | **Addressed:** Standardization of procedures, use of standardized RSI checklist, interventions restricted by scope of practice mentioned as efforts to ensure consistency.  (Methods: Procedures section, Page 6) |
| 10 | Study size | Explain how the study size was arrived at. | - | **Addressed:** Described as a “convenience sample” of 20 PHCPs. No power calculation or formal sample size justification provided. (Methods: Study sample, Page 5-6) |
| 11 | Quantitative variables | Explain how quantitative variables were handled in the analyses. If applicable, describe which groupings were chosen and why. | - | **Addressed:** TI (seconds) analyzed using non-parametric tests due to non-normality. Presented as median and IQR. Grouping by LSI type and PHCP qualification (paramedic vs. physician). (Methods: Data Analysis, Page 9) (Results sections, Figures 1 & 2) |
| 12 | Statistical methods | (a) Describe all statistical methods, including those used to control for confounding. (b) Describe any methods used to examine subgroups and interactions. (c) Explain how missing data were addressed. (d) Cohort study: If applicable, explain how loss to follow-up was addressed. Case-control study: If applicable, explain how matching of cases and controls was addressed. Cross-sectional study: If applicable, describe analytical methods taking account of sampling strategy. (e) Describe any sensitivity analyses. | Clearly indicate the unit of analysis (eg, individual, team, system). Identify repeated measures on subjects, and describe how these issues were addressed. | **Addressed:** Statistical Software: RStudio. Normality: Shapiro-Wilk test, visual inspection. Comparison Methods: Mann-Whitney U test (between groups), Wilcoxon Signed-Rank test (estimated vs. actual TI). Significance: p < 0.05. Unit of Analysis: Implied as LSI performance instance (N=246). Repeated Measures: Not applicable.  how potential non-independence was handled. Missing Data: Not applicable. Confounding Control: Not explicitly addressed via statistical adjustment. (Methods: Data Analysis, Page 9) |
|  | **Results** |  |  |  |
| 13 | Participants | (a) Report the numbers of individuals at each stage of the study—eg, numbers potentially eligible, examined for eligibility, confirmed eligible, included in the study, completing follow-up, and analysed. (b) Give reasons for nonparticipation at each stage. (c) Consider use of a flow diagram. | - | **Addressed:** 20 PHCPs participated (12 paramedics, 8 physicians). Total 246 LSIs performed. Participant flow details beyond total number included not provided. No flow diagram used. Reasons for non-participation not applicable. (Results section, Page 10 and Table 2) |
| 14 | Descriptive data | (a) Give characteristics of study participants (eg, demographic, clinical, social) and information on exposures and potential confounders. (b) Indicate the number of participants with missing data for each variable of interest. (c) Cohort study: Summarize follow-up time (eg, average and total amount). | In describing characteristics of study participants, include their previous experience with simulation and other relevant features as related to the intervention(s). | **Addressed:** Participant characteristics provided: Number by role (paramedic/physician), median years experience, number with prior MCI experience. Previous simulation experience not reported. Missing data not reported. (Results section, Table 2) |
| 15 | Outcome data | Cohort study: Report numbers of outcome events or summary measures over time. Case-control study: Report numbers in each exposure category or summary measures of exposure. Cross-sectional study: Report numbers of outcome events or summary measures. | - | **Addressed:** Median TIs with IQRs reported for all 16 LSIs overall and by PHCP group (where applicable). (Results: Intervention Timings Across Participants section, Figure 1, Figure 2, Table 3) |
| 16 | Main results | (a) Give unadjusted estimates and, if applicable, confounder-adjusted estimates and their precision (eg, 95% confidence intervals). Make clear which confounders were adjusted for and why they were included. (b) Report category boundaries when continuous variables were categorized. (c) If relevant, consider translating estimates of relative risk into absolute risk for a meaningful time period. | For assessments involving >1 rater, interrater reliability should be reported. | **Addressed:** Main results presented as median TIs (unadjusted). Statistical comparisons between groups (Mann-Whitney) and within subjects (Wilcoxon) reported with p-values. Confidence intervals not reported. No adjusted estimates provided. Inter-rater reliability for timing measurement not reported. (Results: Intervention Timings Across Participants section, Figure 2, Table 3) |
| 17 | Other analyses | Report other analyses done—eg, analyses of subgroups and interactions, and sensitivity analyses. | - | **Addressed:** Subgroup analysis by PHCP qualification reported. Analysis of stepwise timings for team-based interventions (RSI, Cricothyrotomy, Chest tube) reported. No interaction or sensitivity analyses reported. (Results: Intervention Timings Across Participants, Results: Team-Based Intervention Timings sections, Figure 2, Figure 3) |
|  | **Discussion** |  |  |  |
| 18 | Key results | Summarise key results with reference to study objectives. | - | **Addressed:** Discussion summarizes key findings: most LSIs < 2 mins, RSI longest, minimal difference between PHCPs, general overestimation of TIs, specific delays in RSI prep/securing. (Discussion section, Page 12) |
| 19 | Limitations | Discuss limitations of the study, taking into account sources of potential bias or imprecision. Discuss both direction and magnitude of any potential bias. | Specifically discuss the limitations of SBR. | **Addressed:** Limitations section explicitly discusses limitations related to the simulation environment (lack of real-world fidelity, distractions, risks), standardization challenges, and convenience sample impacting generalizability. These directly address limitations of SBR. (Discussion: Limitation section, Page 14-15) |
| 20 | Interpretation | Give a cautious overall interpretation of results considering objectives, limitations, multiplicity of analyses, results from similar studies, and other relevant evidence. | - | **Addressed:** Results interpreted cautiously, considering study limitations. Findings compared with previous literature. Potential explanations (e.g., time distortion) discussed. (Discussion section, Page 12-15) |
| 21 | Generalizability | Discuss the generalisability (external validity) of the study results. | Describe generalizability of simulation-based outcomes to patient-based outcomes (if applicable). | **Addressed:** Generalizability discussed primarily in the Limitations section, noting simulation may not fully replicate real-world incidents, patient variability, or different settings. Focus is on generalizability of timings to practice, not directly to patient outcomes. (Discussion: Limitation section, Page 14-15) |
| 22 | Other information: Funding | Give the source of funding and the role of the funders for the present study and, if applicable, for the original study on which the present article is based. | List simulator brand and if conflict of interest for intellectual property exists. | **Addressed:** Funding: Stated “No Funding”. Simulator brands (SIMBODIES Manikin®, Laerdal Manikin®) listed in Methods: Equipment section. No conflicts of interest declared related to simulators. (Declarations: Funding, Page 17) (Methods: Equipment sections, Page 6) |
