# Supplementary material for: Timings of pre-hospital life-saving interventions in mass casualty incidents: an observational simulation study
Source: Scand J Trauma Resusc Emerg Med. 2025 Jun 2;33:100. doi: 10.1186/s13049-025-01417-z (PMC12131418; doi:10.1186/s13049-025-01417-z)
Supplement: Supplementary file 1 — Supplementary Material 1. [file 13049_2025_1417_MOESM1_ESM.zip › Supplementary Material, Table B.docx]

| Table B: Simulation mannequins and equipment used for each life-saving intervention | | | | | |
| --- | --- | --- | --- | --- | --- |
| **Life-saving interventions** | | **SIMBODIES Manikin** | **Laerdal Manikins** | **Equipment** | **Further details** |
| **Airway** | 1. Rapid Sequence Intubation | ✓ |  | Oxygen cylinder + Laryngoscope handle and blades + Suction device with rigid tip + End-tidal carbon dioxide monitor + Endotracheal tube with an inflatable cuff with sterile lubricant + tube tie + Tracheal tube inducer (bougie) + Bag valve mask (BVM) with PEEP valve + Drugs (induction and rocuronium) | **RSI process incorporated 5 Ps**   1. **Pre-preparation**: Equipment preparation and verification using a Pre-RSI challenge-response checklist (see Figure C for details). This ensures readiness of all necessary tools and materials. 2. **Pre-oxygenation**: Simultaneously conducted with the pre-preparation step, typically performed by the clinician responsible for intubation. 3. **Paralysis and Induction**: Initiated following the completion of the checklist, marking readiness for medication administration. This phase includes the administration of Fentanyl 1 mcg/kg, Ketamine 1 mg/kg, and Rocuronium 1 mg/kg. The timing for this phase takes into account that an intravenous line is already established, as IV setup is considered a separate intervention. 4. **Placement with Proof**: Insertion of the Endotracheal Tube (ETT) is timed at around 45 seconds after the medication takes effect. The clinician picks up the laryngoscope and inserts the ETT, ensuring its correct placement. Confirmation is achieved using a stethoscope and capnography. 5. **Postintubation Management**: This final phase involves securing the endotracheal tube with a tube tie to ensure stability and prevent accidental displacement. |
|  | 1. Supraglottic Airway Device (i-gel) | ✓ |  | I-gel Device + Lubricant + BVM | - |
|  | 1. Oral Airway Insertion | ✓ | ✓ | Oropharyngeal airway + BVM | - |
|  | 1. Surgical Cricothyroidotomy |  | ✓ | Scalpel + Cuffed tracheostomy tube + Ten cc syringe + BVM + Tracheal tube inducer (bougie) |  |
| **Breathing** | 1. Oxygen Administration (Non-Rebreather Mask) | ✓ | ✓ | Non-rebreathing masks + Oxygen cylinder |  |
|  | 1. Finger Thoracostomy |  | ✓ | Scalpel + Antiseptic Swabs + Gauze Pads | Performed in conjunction with chest tube. As such, the endpoint of this intervention captures and continues into the subsequent intervention. |
|  | 1. Needle Decompression |  | ✓ | IV needle + Alcohol pad + tape | Performed in the second intercostal space along the midclavicular line |
|  | 1. Chest Tube |  | ✓ | (Same equipment for Finger thoracostomy) + Chest tube with collection device + Scissors + Sutures and needle holder | Performed in the fourth or fifth intercostal space on the anterior axillary line, specifically on the right side. |
| **Circulation** | 1. Intravenous (IV) Access |  | ✓ | IV cannula + Alcohol pads + IV tourniquet + Transparent dressings + Sharps container | IV access simulated in the antecubital fossa.  Performed in conjunction with hemostatic agent. As such, the endpoint of this intervention may capture and continue into the subsequent intervention. |
|  | 1. Analgesia (OFTC) |  | ✓ | Fentanyl lozenge | - |
|  | 1. Intraosseous (IO) Access |  | ✓ | EZ-IO device set + Alcohol swab + Syringe + Sharps container | (IO) access was administered specifically in the proximal tibia area. |
|  | 1. Tourniquet Application (CAT) | ✓ |  | CAT tourniquet | CAT tourniquet was applied on the thigh to simulate a limb-threatening injury. |
|  | 1. Administration Of Tranexamic Acid (IM) | ✓ | ✓ | 2 TXA vials + IM needle + Alcohol pad + Sharps container | Simulated administration in the vastus lateralis muscle areas (one on each side). |
|  | 1. Fluid/Blood Product Transfusions |  | ✓ | (Same equipment for IV access) + IV tubing + Fluid bag + IV stand | - |
|  | 1. Hemostatic Agent (Celox^®^ Gauze)/Wound Packing |  | ✓ | Celox Rapid Hemostatic Gauze + Medical bandage + Scissor | - |
|  | 1. Direct Pressure for Bleeding |  | ✓ | Gauze swab + Dressing | Performed in conjunction with hemostatic agent. As such, the endpoint of this intervention captures and continue into the subsequent intervention. |
